# Supplementary figures and images for: Road surface semantic segmentation for autonomous driving
Source: PeerJ Comput Sci. 2024 Sep 25;10:e2250. doi: 10.7717/peerj-cs.2250 (PMC11623202; doi:10.7717/peerj-cs.2250)

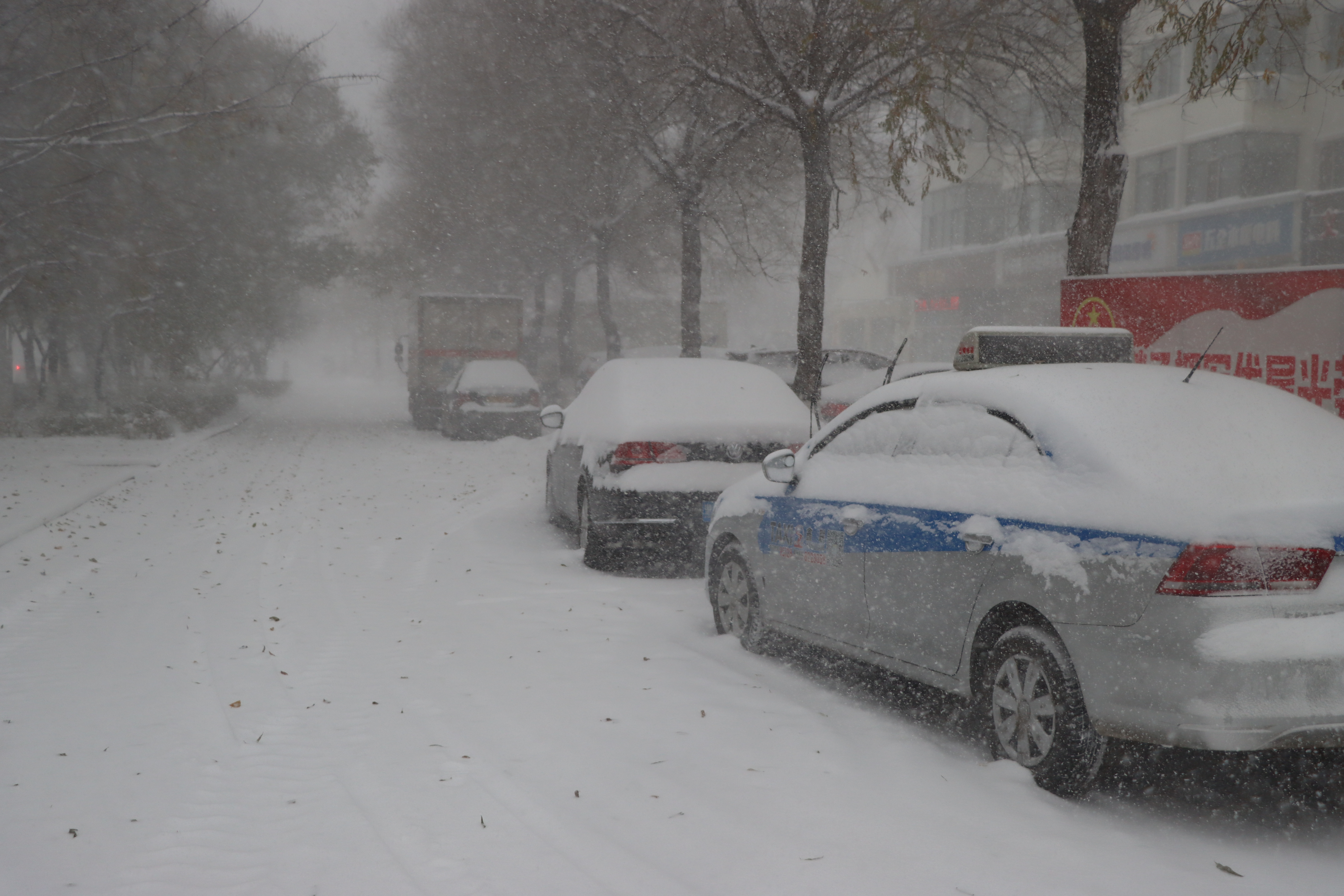

Supplement: Supplemental Information 2 [file peerj-cs-10-2250-s002.zip › data/complex road.jpg]

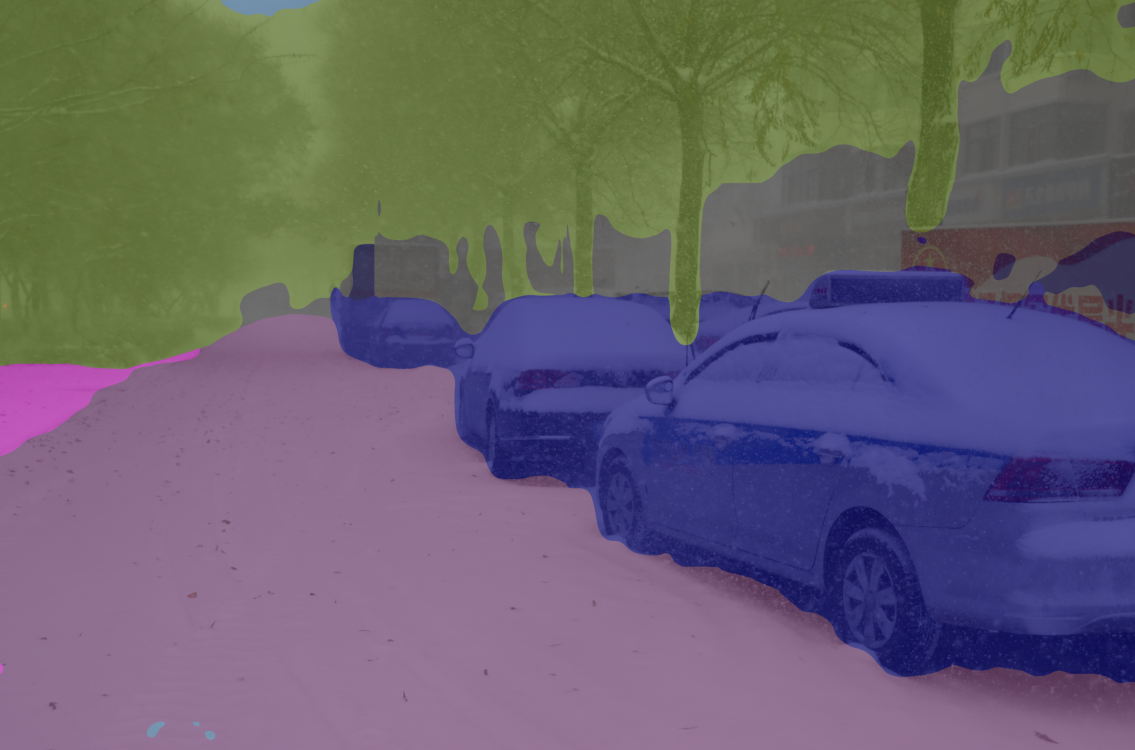

Supplement: Supplemental Information 2 [file peerj-cs-10-2250-s002.zip › data/our method_complex.png]

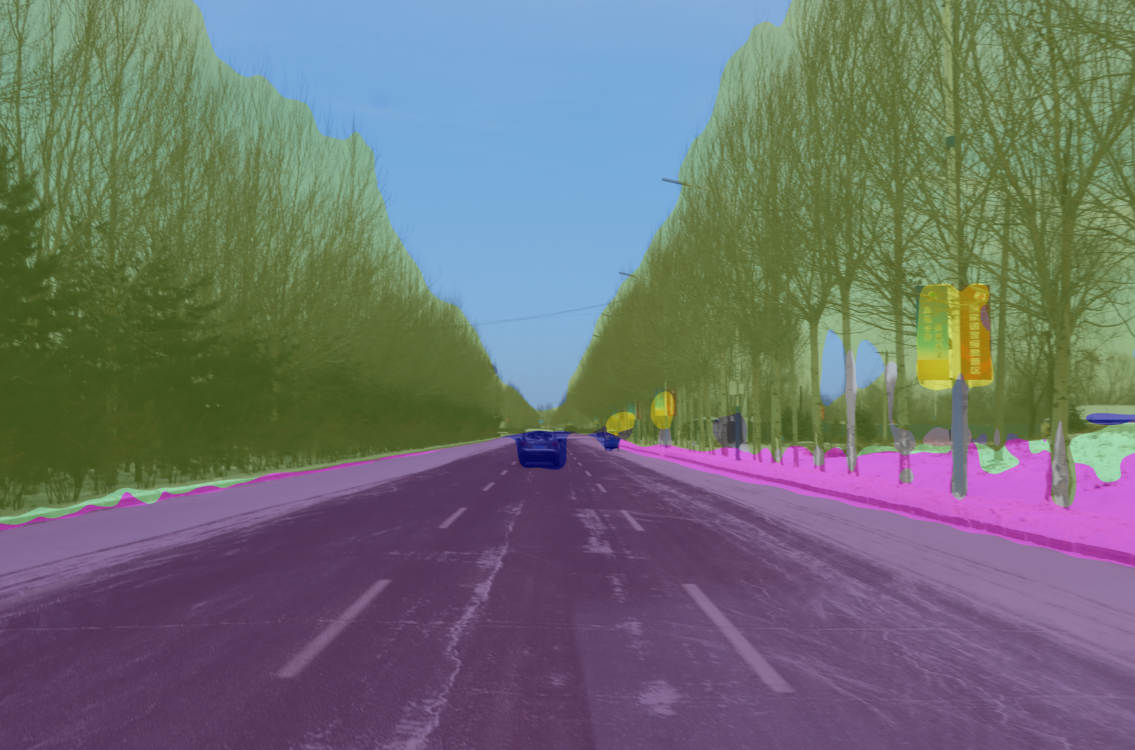

Supplement: Supplemental Information 2 [file peerj-cs-10-2250-s002.zip › data/our method_simple.png]

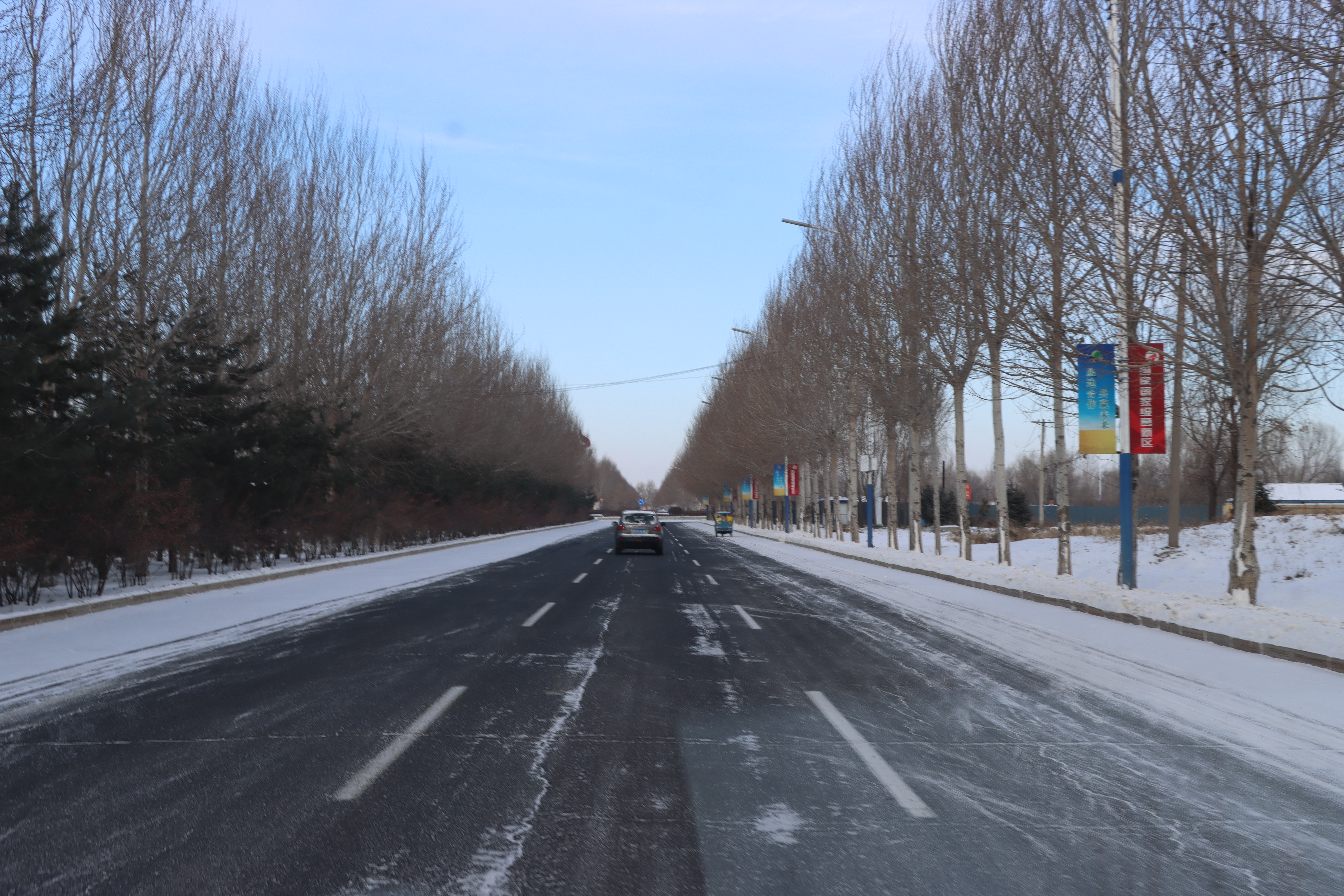

Supplement: Supplemental Information 2 [file peerj-cs-10-2250-s002.zip › data/simple road.jpg]
